# Increased Secondary Attack Rate among Unvaccinated Household Contacts of Coronavirus Disease 2019 Patients with Delta Variant in Japan

**DOI:** 10.3390/ijerph19073889

**Published:** 2022-03-24

**Authors:** Tsuyoshi Ogata, Hideo Tanaka, Yumiko Nozawa, Kazue Mukouyama, Emiko Tanaka, Natsumi Osaki, Etsuko Noguchi, Kayoko Seo, Koji Wada

**Affiliations:** 1 Itako Public Health Center of Ibaraki Prefectural Government, Itako 311-2422, Japan; y.nozawa@pref.ibaraki.lg.jp (Y.N.); k.mukouyama@pref.ibaraki.lg.jp (K.M.); em.tanaka@pref.ibaraki.jp (E.T.); n.oosaki@pref.ibaraki.lg.jp (N.O.); e.noguchi@pref.ibaraki.lg.jp (E.N.); ka.seo@pref.ibaraki.lg.jp (K.S.); 2 Fujiidera Public Health Center of Osaka Prefectural Government, Fujiidera 583-0024, Japan; tanakah61@mbox.pref.osaka.lg.jp; 3 Department of Public Health, Faculty of Medicine and Graduate School of Public Health, International University of Health and Welfare, Tokyo 107-8402, Japan; kwada@iuhw.ac.jp

**Keywords:** COVID-19, SARS-CoV-2, Delta variant, Alpha variant, household transmission, secondary attack rate, unvaccinated, spouse, index patient, Japan

## Abstract

This study aimed to elucidate the household secondary attack rate (HSAR) of the Delta variant in comparison to the Alpha variant, and evaluate the risk factors among unvaccinated household contacts of patients with coronavirus disease 2019 (COVID-19). We studied household contacts of index cases of COVID-19 infected with Delta (L452R mutation), Alpha (N501Y mutation), and wild strain from December 2020 through November 2021 in Itako, Japan. The HSARs of the entire household contact, and the contact of index case with Delta variant were calculated and compared across the risk factors. We used a generalized estimating equation regression model for the multivariate analysis. We enrolled 1257 unvaccinated contacts from 580 households. The HSAR was higher in household contacts of index patients with Delta (48.5%) than with Alpha variant (21.7%) (aOR = 3.34, *p* = 0.000). In Delta variants, the HSAR was higher in household contacts with spousal relationships to index patients (63.4%) than contacts with other relationships (45.5%) (aOR 1.94, *p* = 0.026), and was lower in household contacts of index patients aged ≤19 (33.1%) than for contacts of index cases aged 20–59 years (52.6%) (aOR = 0.50, *p* = 0.027). The result of our study can be used to devise informed strategy to prevent transmission within households.

## 1. Introduction

The Delta variant (phylogenetic assignment of named global outbreak lineage designation B.1.617.2) is a lineage of severe acute respiratory syndrome coronavirus 2 (SARS-CoV-2), the virus causing coronavirus disease 2019 (COVID-19). It was first identified in India in late 2020 and was classified as a variant of concern (VOC) on 11 May 2021 [1]. This variant was associated with an estimated increase in transmissibility of 97% [2]. SARS-CoV-2 VOCs bearing the L452R spike protein mutation demonstrate increased transmissibility, infectivity, and avoidance of antibody neutralization [3]. As of 12 November, the Delta variant cases were reported in 191 countries across all six WHO regions. Furthermore, the Delta variant has become the dominant strain in Japan and many other countries until the emergence of Omicron variant [4]. 

Japan has experienced a surge of COVID-19 cases six times: the first three waves with wild strain, the fourth wave with Alpha variant that peaked in May 2021, the fifth wave with Delta variant that peaked in August 2021, and the sixth wave with the ongoing Omicron variant. Cases of domestic transmission of Delta-variant was confirmed in the latter half of May 2021 [5]. The fifth wave of COVID-19, which was primarily caused by the Delta variant, occurred from the latter half of July 2021 to August 2021 [6,7].

The secondary attack rates of household contacts for COVID-19 are important in assessing the transmissibility of SARS-CoV-2, and the risk factors for infectivity of index patients, as well as the susceptibility of contacts [8]. A meta-analysis estimated the household secondary attack rate (HSAR) to be at 18.9%, which was higher among contacts with spousal relationships and adult contacts [9].

While studies regarding HSAR of COVID-19 due to wild strain have been published in various countries, studies reporting HSAR of Delta SARS-CoV-2 variant remains sparse [10,11,12,13,14,15]. HSAR was 26% for Delta variant in Singapore, and 38% for Alpha variant in Denmark [10,16]. Although the Omicron variant is the current predominant strain, we believe that studying the transmissibility the Delta variant compared to Alpha variant and its risk factors are useful for understanding mechanism of viral replacement and intervention against both the Omicron and Delta variants.

In this study, we specifically attempted to address several questions. First, in Japan, the Delta variant emerged following the Alpha variant. Therefore, the present study tried to determine whether the HSAR of COVID-19 patients with Delta strain was higher than that of patients with the Alpha variant. A previous study reported higher HSAR among Delta variant cases compared to alpha cases [17].

Second, the present study attempted to identify the risk factors for infection transmission of Delta variant from index patients to their close contacts. Previous studies on wild strains reported higher HSARs for spouse contact [18,19,20,21,22,23], household contacts of young index patient [24], and diagnostic delay [23,24]. The present study aimed to determine whether the HSAR for unvaccinated spouse contacts, contacts of index patients with young age, and with long diagnostic delay were also high in the Delta variant.

Third, understanding the trajectory of HSAR is also important for the evaluation of an epidemic. Since the number of infections of the fifth wave continuously declined after September in Japan, the present study aimed to compare HSAR for the Delta strain before and after the peak of the fifth wave in August.

Although comparison of HSAR between vaccinated and unvaccinated persons is important, the proportion of vaccinated contacts was small except the aged during Delta dominant fifth wave in Japan.

This study aimed to elucidate the HSARs of the Delta variant in comparison with the Alpha strain and assess the risk factors among unvaccinated household contacts of patients with COVID-19.

## 2. Materials and Methods

### 2.1. Study Design

The study used an observational study design.

### 2.2. Setting

This study was conducted in the jurisdiction of the Itako Public Health Center (PHC) in Japan. The jurisdictional area is located 80 km from Tokyo, and has a total population of approximately 265,000.

### 2.3. Index COVID-19 Cases

The index COVID-19 cases in this study were individuals living in the jurisdiction with confirmed SARS-CoV-2 infection, as defined by the Itako PHC, from December 2020 through November 2021. The number of confirmed COVID-19 patients living in the jurisdiction of the Itako PHC was 2454 (0.9%) at the end of November 2021.

In Japan, according to the Infectious Diseases Control Law (The Law; No. 104 in 1998), the public health center must be notified of all COVID-19 cases [25]. SARS-CoV-2 infections for the index patients were mainly confirmed using polymerase chain reaction (PCR) tests with a cycle threshold value of 40. Part of them were confirmed by antigen quantitative tests, monoclonal antigen qualitative tests, loop-mediated isothermal amplification tests (LAMP), or Nicking Enzyme Amplification Reaction (NEAR) by clinicians. The PCR test was performed if the results of any of the other tests were ambiguous.

The public health center implemented an epidemiological investigation of the patients based on the Law. The nurses of the public health center interviewed the patients and collected data on demographics, symptoms, and history of confirmed contact with a COVID-19 patient.

We defined patients with COVID-19 with apparent exposure to SARS-CoV-2 outside household as the index case in the household. If no patient with COVID-19 had a history of exposure to SARS-CoV-2 outside household and several patients with COVID-19 in a household were symptomatic, the COVID-19 patient who had the earliest symptom onset date, either vaccinated or unvaccinated, was defined as the index case in a household, and other members in the household were included as participant contacts. Households with two members having the same earliest onset date were excluded from the analysis.

### 2.4. Participant Household Contacts

The participants eligible for this study were the unvaccinated household contacts of index patients with COVID-19, who are living with the patient, and are usually sleeping in the same house. Since most of the contacts of patients with wild strain and Alpha variant were unvaccinated, and the proportion of vaccinated contacts among non-elderly contacts with the Delta variant was also relatively small, we excluded vaccinated contacts from the analysis. Unvaccinated was defined as a zero vaccination. If an index case had no household contact, the household was excluded from the analysis. As the number of confirmed COVID-19 cases per population in the jurisdiction of the Itako PHC was 0.9% at the end of November 2021, we assumed that household contacts were susceptible to SARS-CoV-2 infection.

The PHC implements a law-based bidirectional contact tracing of patients, whether symptomatic or not [25]. Information on breakup of household contact’s comorbid illness had not been recorded. Based on the regulations on infectious diseases, Itako PHC collected PCR test samples on all household contacts of index cases. If a contact had a negative PCR test result but had a new symptom onset, another PCR test was performed as confirmatory test.

### 2.5. Variant Type of SARS-CoV-2 Strains of Participant Contacts

Among unvaccinated COVID-19 contacts, we defined contact of the index case reported up to March 19 as contact with wild type strain cases. In Ibaraki prefecture, the N501Y mutation was not found in the tests until the 11th week of 2021, and thus, the wild strains were selected from the participants tested by the 11th week of 2021. The first cases of virus variants, Alpha variant, were detected between 22 and 28 March 2021 (the 12th week) in Ibaraki [26].

Contact with the Alpha strain was defined as contact of the index patient with positive results for N501Y mutation until 20 June 2021 or negative results for L452R mutation after 21 June 2021 among the patient, or the patient’s contacts. Almost all cases with the N501Y mutation during the study period in Japan were confirmed to be the Alpha variant by RNA sequencing. The proportion of N501Y mutation was 98.7% in the 23rd week (7–13 June 2021), and the first L452R mutation was detected in the 25th week (21–27 June 2021) in Ibaraki [26].

Among unvaccinated COVID-19 contacts, we defined contact of the index case with the L452R variant among the patients or their contacts as contacts of the Delta variant. In Japan, screening for the L452R mutation was implemented in approximately 40–50% of samples from July 2021. The L452R mutation is also found in other variant strains of interest, such as the B.1.617.1 (Kappa) variant. However, almost all cases with the L452R mutation in Japan have been confirmed to be the Delta variant by RNA sequencing. In particular, the domestic number of VOCs confirmed by genome sequencing was 42,721 for B.1.617.2 (Delta), 47,856 for B.1.1.7 (Alpha), and eight for B.1.617.1 (Kappa) as of September 27 [6].

If the index case was reported after 22 March, and both N501Y and L452R mutation screening were not performed, or not detected for the index patient and contacts, the contact was excluded from the study.

### 2.6. Outcome, Data Collection, and Variables

The outcome of interest in this study was SARS-CoV-2 transmission to household contacts of index COVID-19 cases. HSAR was defined as proportion of SARS-CoV-2 transmission among household contacts.

Household contacts were interviewed by public health nurses. Through bidirectional contact tracing after SARS-CoV-2 confirmation, physicians and nurses of the Itako PHC collected the participants’ demographic data, date of symptom onset, and behaviors prior to testing [25].

### 2.7. Statistical Analysis

We described the flow of enrollment and the characteristics of index patients with COVID-19 and household contacts.

The HSARs in all household contacts were calculated and compared across risk factors of index cases, household contacts, and viral type. Data are presented as proportions with percentages and 95% confidence intervals (CIs). For multivariate analyses, we used a generalized estimating equation (GEE) regression model to adjust for confounding by household cluster and calculated the adjusted odds ratio (aOR) and *p*-value.

The HSARs in household contacts of the Delta variant were also calculated and compared across risk factors of index cases, household contacts, and sample collection time. Age was classified as ≤19 (child and adolescence), 20–59 (adult), and ≥60 (elders). The HSAR of the Delta variant was compared among contacts of index patient during 25–31 weeks when they coexisted with the Alpha variant, index patient during 32–34 weeks when the peak was seen, and index patient after 35 weeks when the infection decreased.

As elder contacts were vaccinated for patients with Delta variants and were not vaccinated enough for patients with wild strain and Alpha variant in Japan, we implemented sensitive analyses by comparing crude HSAR by virus variant among unvaccinated contacts with age ≤59.

Statistical analyses were performed using R (version 4.4-1; R Foundation for Statistical Computing, Vienna, Austria). Statistical significance was defined as *p* < 0.05.

## 3. Results

Households with a size of not less than two was screened from December 2020 through November 2021, and households without viral strain classification and contacts vaccinated at least once were excluded. Table 1 shows period, flow of enrolment of index cases, including test for confirmation, and unvaccinated household contacts. SARS-CoV-2 infections for 76% of index patients and all of contacts were confirmed using PCR test. We enrolled 1257 household contacts in 580 households (Table 1 and Table 2).

Table 3 shows the prevalence of SARS-CoV-2 infection among unvaccinated household contacts. In total, 390 of 1257 household contacts were infected with SARS-CoV-2, with an overall HSAR of 31.0%.

The HSAR was higher for household contacts with spousal relationships to index COVID-19 patients (47.0%) compared to contacts with other relationships (28.6%) (aOR 1.49, *p* = 0.022). The HSAR was lower for household contacts of index patients aged less than 20 years (26.9%) than for contacts of index cases aged between 20 and 59 years (32.2%) (aOR = 0.51, *p* = 0.006). The HSAR was higher for household contacts of index patients with ≥3 days of diagnostic delay (35.1%) than for contacts of index cases with ≤2 days of diagnostic delay (29.6%), but the difference was not significant (*p* = 0.051).

The HSAR was higher for household contacts of index patients with Delta variant (48.5%) than for contacts of index cases with Alpha variant (21.7%) (aOR = 3.34, *p* = 0.000).

When the multivariate analysis was implemented with the wild type as reference, the HSAR was higher for household contacts of index patients with Delta variant (48.5%) than for contacts of index cases with wild type (17.9%) (aOR = 4.26, *p* = 0.00).

Table 4 shows the prevalence of SARS-CoV-2 infection in 503 unvaccinated household contacts of the index patient with the Delta variant. In total, 244 household contacts were infected with SARS-CoV-2; the overall HSAR was 48.5%.

The HSAR was higher for household contacts with spousal relationships to index COVID-19 patients (63.4%) than for contacts with other relationships (45.5%) (aOR 1.94, *p* = 0.026). The HSAR was lower for household contacts of index patients aged less than 20 years (33.1%) than for contacts of index cases aged between 20 and 59 years (52.6%) (aOR = 0.50, *p* = 0.027). The HSAR was higher for household contacts of index patients with ≥3 days of diagnostic delay (58.1%) than for contacts of index cases with ≤2 days of diagnostic delay (45.1%), but the difference was not significant (*p* = 0.051).

The HSAR was not significantly different among the contacts in the three periods for sample collection in the index patients. The HSAR was not significantly different for household contacts of vaccinated index patients (58.8%) compared with contacts of unvaccinated index cases (47.3%).

Table 5 shows the prevalence of SARS-CoV-2 infection among household contacts aged ≤59 years by virus strain as a result of sensitive analyses. The crude HSAR was higher for household contacts of index patients with Delta variant than for contacts of index cases with Alpha and wild type.

## 4. Discussion

The secondary attack rate among unvaccinated household contacts of COVID-19 was 31% in Itako, Japan, from December 2020 through November 2021. The HSAR of unvaccinated contacts of the index patient with the Delta variant was 48%. Previous studies reported that HSAR among unvaccinated, or mainly unvaccinated households were 26% in Singapore [10], 35% in Thailand [11], 38% and 28% in Korea [12,13], 53% in USA [14], and 22% for household of unvaccinated index patients in Netherland [15]. The HSAR in the present study is relatively similar to the previous studies.

The HSAR was higher for unvaccinated household contacts of index patient with Delta variant (48%) than for unvaccinated contacts of index cases with Alpha variant (22%) (aOR = 3.3, *p* = 0.000). Among unvaccinated contacts aged ≤59 years, the crude HSAR in the Delta variant was 48%, which was significantly higher than the 24% in the Alpha variant. In a previous study, the adjusted odds ratio of household transmission was 1.70 among Delta variant cases compared to Alpha variant cases in England [17], which was corroborated by the result of the present study. In both England and Japan, the Delta variant replaced the Alpha variant, which can be attributed to the increased transmissibility of the Delta variant compared with the Alpha variant. It is necessary to compare HSAR between Omicron variant and Delta variant among both unvaccinated and vaccinated people since Omicron variant has significantly surpassed Delta variant.

In the Delta variant, the HSAR for contacts with a spousal relationship was 63%, which was significantly higher than the 45% for non-spousal contacts. To the best of our knowledge, no studies have reported higher HSAR for spousal contacts of the Delta variant. This result was consistent with the findings of several previous studies reporting a higher HSAR for spouses among contacts of patients with wild strains [18,19,20,21,22,23]. The spousal contact and the index case may spend longer periods of time together within the same household compared with other household members.

In the Delta variant, the HSAR was significantly higher in unvaccinated contacts of index patients aged ≤19 years compared with contacts of index patients age 20–59. Prevention of transmission from children and young people may be especially important for preventing spread of transmission in the community. A previous study reported higher HSAR for households of young index patients with wild strains [24]. The reason of higher HSAR in contacts of index cases younger than 20 years is not apparent. Differences of viral shedding, proximity to other households, staying period in the household among age groups might be related to it, and are necessary to be studied.

In Delta variants, the HSAR was 45% for unvaccinated household contacts of index patients with ≤2 days of diagnostic delay, and lower than 58% for contacts of index patients with ≥3 days of diagnostic delay; however, the difference was not significant. In previous studies in Japan, the long diagnostic delay of COVID-19 index patients was associated with a high HSAR for household contacts [23,24]. A study using a mathematical model showed that the delay between symptom onset and isolation played a major role in controlling the COVID-19 outbreak [27].

There was no change in the HSAR of the Delta variant over time, although the fifth wave of COVID-19 in Japan, mainly caused by the Delta variant, declined continuously after September 2021 [6,7].

The present study was implemented by the governmental body in charge of all COVID-19 cases in the jurisdiction. As the cumulative incidence of confirmed COVID-19 per population in the jurisdiction was low during the study period, we assumed that household contacts were susceptible to infection.

This study had several limitations. First, we basically performed the PCR test only once unless an asymptomatic contact became symptomatic during quarantine period, potentially missing infected contacts. Second, the associated household environmental factors, including the level of crowding, lifestyle, precaution measures of each contact, and proximity of contacts to the index cases, were not evaluated. Third, we defined the patient with the earliest onset date as the index case in a household without any COVID-19 cases with apparent exposure to SARS-CoV-2. It is possible that the index cases might have been misclassified as secondary cases. Last, the Delta and Alpha variants were mainly confirmed by the L452R and N501Y mutations, respectively. However, genome sequencing revealed a coincidence between the mutation and the variant in Japan; the mutations could substitute the viral strains [6].

Further studies are necessary to analyze the association between HSAR and factors, such as variants of the virus, vaccination status, environmental factors, and other risk factors of index patient and household contact. 

It is also necessary to continue surveillance of epidemiological data and other VOCs, including Omicron variants.

## 5. Conclusions

The HSAR was higher in unvaccinated household contacts of index patients with Delta variant than for contacts of index cases with Alpha variant. In the Delta variant, the HSAR was higher in unvaccinated household contacts with spousal relationships, and was lower in household contacts of index patients aged <20 years than for contacts of index cases aged between 20 and 59 years.

## Figures and Tables

**Table 1:** Period, flow of enrollment of index cases, including test for confirmation, and house-hold contacts.

Period	28 March–30 November 2020	1 December–19 March 2021	20 March–14 April 2021	15 April–20 June 2021	21 June–30 November 2021	Total
Pandemic wave in Japan	The 1st and 2nd	The 3rd		The 4th	The 5th	
No. of all index patients	92	179	28	171	1168	
Enrolled as wild strain		All				
Number of index patient		179				179
Unvaccinated household		459				459
Enrolled as Alpha variant				N501Y (+)	L452R (−)	
Number of index patient				60	63	123
Unvaccinated household				176	119	295
Enrolled as Delta variant					L452R (+)	
Number of index patient					278	278
Unvaccinated household					503	503
Number of total enrolled index patients	0	179	0	60	341	580
					
Test for confirmation						
PCR		149		51	238	438
Antigen test		23		8	85	116
LAMP		7		1	14	22
NEAR					2	2
Unknown					2	2

LAMP; loop-mediated isothermal amplification tests; NEAR; Nicking Enzyme Amplification Reaction; N501Y (+); N501Y mutation positive; L452R (−); L452R mutation negative; L452R (+); L452R mutation positive.

**Table 2:** Characteristics of COVID-19 patients, and unvaccinated household contacts.

Variables	Index COVID-19 Cases	Household Contacts
*N*	580	1257
Relationship to index patient		
Spouse		168 (13.3%)
Other		1089 (86.6%)
Diagnostic delay from onset		
≤2 days	390 (67.2%)	
≥3 days	173 (29.8%)	
Asymptomatic	17 (2.9%)	
Sex		
Male	322 (55.5%)	607 (48.3%)
Female	258 (44.5%)	650 (51.7%)
Age		
≤19	112 (19.3%)	471 (37.4%)
20–59	406 (70.0%)	601 (47.8%)
≥60	62 (10.7%)	185 (14.7%)

**Table 3:** Secondary attack rate among unvaccinated household contacts of COVID-19 patients by factors.

Variables	Household Contacts	Infected Contacts	Secondary Attack Rate	Multivariate Analysis
			% (95% CI)	aOR (*p*-Value)
*N*	1257	390	31.0% (28.5–33.6)	
Risk factors in household contacts				
Relationship to index patient				
Spouse	168	79	47.0% (39.6–54.6)	1.49 (0.022)
Other	1089	311	28.6% (26.0–31.3)	1
Sex				
Male	607	179	29.5% (26.0–33.2)	0.85 (0.095)
Female	650	211	32.5% (29.0–36.2)	1
Age				
≤19	471	150	31.8% (27.6–36.2)	0.93 (0.56)
20–59	601	186	30.9% (27.4–43.5)	1
≥60	185	54	29.2% (23.1–36.1)	1.14 (0.40)
Risk factors in index COVID-19 cases				
Diagnostic delay from onset				
≤2 days	825	244	29.6% (26.6–32.8)	1
≥3 days	410	144	35.1% (30.7–39.9)	1.41 (0.051)
Asymptomatic	22	2	9.1% (1.5–29.3)	0.15 (0.074)
Sex				
Male	702	220	31.3% (28.0–34.9)	0.99 (0.96)
Female	555	170	30.6% (26.9–34.6)	1
Age				
≤19	208	56	26.9% (21.4–33.4)	0.51 (0.006)
20–59	949	306	32.2% (29.3–35.3)	1
≥60	100	28	28.0% (20.1–37.6)	1.11 (0.72)
Viral type				
Wild type	459	82	17.9% (14.6–21.7)	0.80 (0.37)
Alpha	295	64	21.7% (17.4–26.8)	1
Delta-	503	244	48.5% (44.2–52.9)	3.339 (0.000)

All variables were included in the analysis.

**Table 4:** Secondary attack rate among unvaccinated household contacts of COVID-19 patients with Delta variant.

Variables	Household Contacts	Infected Contacts	Secondary Attack Rate	Multivariate Analysis
			% (95% CI)	aOR (*p*-Value)
*N*	503	244	48.5% (44.2–52.9)	
Risk factors in household contacts				
Relationship to index patient				
Spouse	85	54	63.4% (52.9–73.0)	1.94 (0.026)
Other	418	190	45.5% (40.7–50.2)	1
Sex				
Male	242	110	45.5% (39.3–51.8)	0.71 (0.55)
Female	261	134	51.3% (45.3–57.3)	1
Age				
≤19	239	115	48.1% (41.9–54.4)	1.33 (0.19)
20–59	230	109	47.4% (41.0–53.8)	1
≥60	34	20	58.8% (42.2–73.6)	1.65 (0.19)
Risk factors in index COVID-19 cases				
Diagnostic delay from onset				
≤2 days	337	152	45.1% (39.9–50.4)	1
≥3 days	155	90	58.1% (50.2–65.5)	1.66 (0.051)
Asymptomatic	11	2	18.2% (4.3–49.0)	0.23 (0.18)
Sex				
Male	267	129	48.3% (42.4–54.3)	0.98 (0.92)
Female	236	115	48.7% (42.4–55.1)	1
Age				
≤19	121	40	33.1% (25.3–41.9)	0.50 (0.027)
20–59	365	192	52.6% (47.5–57.7)	1
≥60	17	12	70.6% (46.5–86.8)	1.87(0.22)
Week of collecting sample				
25–31	165	80	48.5% (41.0–56.1)	1
32–34	251	128	50.1% (44.8–57.1)	1.36 (0.25)
35–	87	36	41.4% (31.6–51.9)	0.86 (0.70)
	Vaccination			
None	452	214	47.3% (42.8–52.0)	1
1–2	51	30	58.8% (45.1–71.2)	1.30 (0.49)

All variables were included in the analysis. CI = confidence interval.

**Table 5:** Secondary attack rate among unvaccinated household contacts with age ≤59 by virus type.

Variables	Household Contacts	Infected Contacts	Secondary Attack Rate
			% (95% CI)
*N*	1104	354	32.1% (29.4–34.9)
Total			
Wild type	345	51	14.8% (11.4–19.0)
Alpha	258	61	23.6% (18.9–29.2)
Delta	501	242	48.3% (44.0–52.7)

CI = confidence interval.

## Data Availability

The data presented in this study are available upon reasonable request from the corresponding author. The data are not publicly available because of the protection of personal information.

## References

[1] World Health Organization Statement on the Second Meeting of the International Health Regulations (2005) Emergency Committee Regarding the Outbreak of Novel Coronavirus (2019-nCoV). https://www.who.int/news-room/detail/30-01-2020-statement-on-the-second-meeting-of-the-international-health-regulations-(2005)-emergency-committee-regarding-the-outbreak-of-novel-coronavirus-(2019-ncov).

[2] Campbell F., Archer B., Laurenson-Schafer H., Jinnai Y., Konings F., Batra N., Pavlin B., Vandemaele K., Van Kerkhove M.D., Jombart T. (2021). Increased transmissibility and global spread of SARS-CoV-2 variants of concern as at June 2021. Eurosurveillance.

[3] Deng X., Garcia-Knight M.A., Khalid M.M., Servellita V., Wang C., Morris M.K., Sotomayor-González A., Glasner D.R., Reyes K.R., Gliwa A.S. (2021). Transmission, infectivity, and neutralization of a spike L452R SARS-CoV-2 variant. Cell.

[4] World Health Organization Weekly Epidemiological Update on COVID-19–13. https://www.who.int/publications/m/item/weekly-epidemiological-update-on-covid-19---13-october-2021.

[5] National Institute of Infectious Diseases Current Situation of Infection. https://www.niid.go.jp/niid/en/2019-ncov-e/10415-covid19-ab36th-en.html.

[6] COVID-19 Advisory Board of the Ministry of Health, Labor and Welfare Current Situation of Infection and Others. https://www.mhlw.go.jp/stf/seisakunitsuite/bunya/0000121431_00294.html.

[7] Ministry of Health, Labor and Welfare Visualizing the Data: Information on COVID-19 Infections. https://covid19.mhlw.go.jp/en/.

[8] Madewell Z.J., Yang Y., Longini I.M., Halloran M.E., Dean N.E. (2020). Household transmission of SARS-CoV-2: A systematic review and meta-analysis. JAMA Netw. Open.

[9] Madewell Z.J., Yang Y., Longini I.M., Halloran M.E., Dean N.E. (2021). Factors associated with household transmission of SARS-CoV-2: An updated systematic review and meta-analysis. JAMA Netw. Open.

[10] Ng O.T., Koh V., Chiew C.J., Marimuthu K., Thevasagayam N.M., Mak T.M., Chua J.K., Ong S.S., Lim Y.K., Ferdous Z. (2021). Impact of Delta variant and vaccination on SARS-CoV-2 secondary attack rate among household close contacts. Lancet Reg. Health-West Pac..

[11] Watanapokasin N., Siripongboonsitti T., Ungtrakul T., Muadchimkaew M., Wongpatcharawarakul S., Auewarakul C., Mahanonda N. (2021). Transmissibility of SARS-CoV-2 variants as a secondary attack in Thai households: A retrospective study. IJID Reg..

[12] Yi S., Kim J.M., Choe Y.J., Hong S., Choi S., Ahn S.B., Kim M., Park Y.J. (2022). SARS-CoV-2 Delta Variant Breakthrough Infection and Onward Secondary Transmission in Household. J. Korean Med. Sci..

[13] Hwang H., Lim J.S., Song S.A., Achangwa C., Sim W., Kim G., Ryu S. (2022). Transmission dynamics of the Delta variant of SARS-CoV-2 infections in South Korea. J. Infect. Dis..

[14] Dougherty K., Mannell M., Naqvi O., Matson D., Stone J. (2021). SARS-CoV-2 B. 1.617. 2 (Delta) variant COVID-19 outbreak associated with a gymnastics facility—Oklahoma, April–May 2021. Morb. Mortal. Wkly. Rep..

[15] de Gier B., Andeweg S., Backer J.A., Hahné S.J., van den Hof S., de Melker H.E., Knol M.J., RIVM COVID-19 Surveillance and Epidemiology Team (2021). Vaccine effectiveness against SARS-CoV-2 transmission to household contacts during dominance of Delta variant (B.1.617.2), The Netherlands, August to September 2021. Eurosurveillance.

[16] Lyngse F.P., Mølbak K., Skov R.L., Christiansen L.E., Mortensen L.H., Albertsen M., Møller C.H., Krause T.G., Rasmussen M., Michaelsen T.Y. (2021). Increased transmissibility of SARS-CoV-2 lineage B.1.1.7 by age and viral load. Nat. Commun..

[17] Allen H., Vusirikala A., Flannagan J., Twohig K.A., Zaidi A., Chudasama D., Lamagni T., Groves N., Turner C., Rawlinson C. (2021). Household transmission of COVID-19 cases associated with SARS-CoV-2 Delta variant (B.1.617.2): National case-control study. Lancet Reg. Health Eur..

[18] Shah K., Saxena D., Mavalankar D. (2020). Secondary attack rate of COVID-19 in household contacts: A systematic review. QJM Int. J. Med..

[19] Li W., Zhang B., Lu J., Liu S., Chang Z., Peng C., Liu X., Zhang P., Ling Y., Tao K. (2020). Characteristics of Household Transmission of COVID-19. Clin. Infect. Dis..

[20] Wu J., Huang Y., Tu C., Bi C., Chen Z., Luo L., Huang M., Chen M., Tan C., Wang Z. (2020). Household transmission of SARS-CoV-2, Zhuhai, China, 2020. Clin. Infect. Dis..

[21] Liu T., Liang W., Zhong H., He J., Chen Z., He G., Song T., Chen S., Wang P., Li J. (2020). Risk factors associated with COVID-19 infection: A retrospective cohort study based on contacts tracing. Emerg. Microbes Infect..

[22] Chaw L., Koh W.C., Jamaludin S.A., Naing L., Alikhan M.F., Wong J. (2020). SARS-CoV-2 transmission in different settings: Analysis of cases and close contacts from the Tablighi cluster in Brunei Darussalam. Emerg. Infect. Dis..

[23] Ogata T., Irie F., Ogawa E., Ujiie S., Seki A., Wada K., Tanaka H. (2021). Secondary attack rate among non-spousal household contacts of coronavirus disease 2019 in Tsuchiura, Japan, August 2020–February 2021. Int. J. Environ. Res. Public Health.

[24] Miyahara R., Tsuchiya N., Yasuda I., Ko Y.K., Furuse Y., Sando E., Nagata S., Imamura T., Saito M., Morimoto K. (2021). Familial clusters of coronavirus disease in 10 prefectures, Japan, February–May 2020. Emerg. Infect. Dis..

[25] National Institute of Infectious Diseases, Infectious Disease Epidemiology Center Manual Conducting for Active Epidemiological Surveillance of Patients with Novel Coronavirus Infection (Provisional Version on May 29). May 2020. https://www.niid.go.jp/niid/ja/diseases/ka/corona-virus/2019-ncov/2484-idsc/9357-2019-ncov-02.html.

[26] Institute of Health of Ibaraki Prefectural Government Situation of Tests on Variant Virus of SARS-CoV-2. https://www.pref.ibaraki.jp/hokenfukushi/eiken/kikaku/covid-19_ibarakieiken_kensa.html.

[27] Hellewell J., Abbott S., Gimma A., Bosse N.I., Jarvis C.I., Russell T.W., Munday J.D., Kucharski A.J., Edmunds W.J., Centre for the Mathematical Modelling of Infectious Diseases COVID-19 Working Group (2020). Feasibility of controlling COVID-19 outbreaks by isolation of cases and contacts. Lancet Glob. Health.

